# The Effect of Renal Transplantation on Ventricular Repolarization in Children with Chronic Renal Failure

**Published:** 2013-11-01

**Authors:** H. Amoozgar, A. Tavakoli, M. H. Fallahzadeh, A. Derakhshan, M. Basiratnia

**Affiliations:** 1 *Division of Pediatric Cardiology, *; 2 *Department of Pediatric, Namazi Hospital, Shiraz, Iran*; 3 *Division of Pediatric Nephrology, Department of Pediatric, Namazi Hospital, Shiraz, Iran*

**Keywords:** Child, Electrocardiography, Kidney transplantation, QT interval, QT dispersion, T-end, Ventricular repolarization

## Abstract

Background: Chronic renal failure and hemodialysis affect many ECG parameters which can affect cardiac repolarization.

Objective: To investigate the change in ventricular repolarization before and after kidney transplantation in children.

Methods: A total of 45 children with end-stage renal disease, 45 children at least 6 months after successful renal transplantation, and 45 normal age-matched subjects were enrolled into this study. A 12-lead ECG was recorded in the 3 groups. QT dispersion, QTc dispersion, and T peak to T end (TPE) dispersion were measured.

Results: In the patients before and after renal transplantation and the normal children, respectively, the mean±SD QT dispersion was 0.083±0.033, 0.056±0.029, and 0.033±0.016 (p<0.01); the mean±SD QTc dispersion was 0.104±0.038, 0.066±0.033, and 0.039±0.020 (p<0.01); the mean±SD TPE interval dispersion was 0.060±0.021, 0.045±0.021, and 0.034±0.019 (p<0.01). There was a significant correlation between left intra-ventricular diastolic diameter and QT dispersion, QTc dispersion, and TPE dispersion. The systolic velocity of the mitral valve also correlated with TPE dispersion (r=0.44, p=0.01).

Conclusion: In children with chronic renal failure, indices of ventricular repolarization improve after transplantation, though they still remain longer than the normal values.

## INTRODUCTION

The leading cause of death in patients with chronic renal failure is premature cardiovascular disease. Although the adult patients have many risk factors such as hypertension and hypercholesterolemia, pediatric patients are less likely to develop these risk factors [1-4].

The pattern of cardiovascular involvement in patients with chronic kidney disease differs from that in the general population; these patients have increased chance of sudden death rather than myocardial infarction. The increased QT interval in ECG in these patients, is associated with the development of arrhythmia and sudden cardiac death [5]. Although QT dispersion increases in adult patients with chronic renal failure who are under hemodialysis, it becomes the normal after renal transplantation [6]. 

QTc, QTc dispersion, and QT dispersion are risk factor for the development of arrhythmia in the patients under chronic hemodialysis [7]. QT dispersion is an indicator of general abnormality of ventricular repolarization; it reflects regional differences in ventricular recovery time and has been linked to the concurrent malignant arrhythmia in various cardiac diseases [8, 9]. QT dispersion is significantly longer in patients under chronic hemodialysis compared with the healthy individuals; it prolongs after the hemodialysis, as well [10].

Other indices of ventricular repolarization are “T peak to T end” (TPE) interval and dispersion. TPE interval provides an index of maximum dispersion of repolarization and reflects the transmural dispersion of the repolarization vector among different regions of the ventricle [11]. The shape of the T wave is as important as the duration of the QT interval itself. Changes in T wave morphology are associated with increased repolarization of the myocardium [12]. TPE interval shows ventricular repolarization and is an indicator of arrhythmia.

We conducted this study to determine the effect of renal transplantation on the QT dispersion, QTc dispersion, and TPE dispersion in pediatric patients with chronic renal failure.

## PATIENTS AND METHODS

This study was conducted from September 2009 to May 2011, in Namazi Hospital, Shiraz, Iran, on a group of patients aged <18 years. The study was performed on 45 pediatric patients with ESRD who were candidate for renal transplantation, none of whom had any signs of heart failure or electrolyte abnormalities; 45 consecutive kidney transplant recipients with functioning graft patients at least six months after a successful renal transplantation; and 45 healthy children <18 years old, who had no cardiac or other organic diseases. For each patient, serum level of sodium, potassium, and calcium was checked; a 12-lead digital ECG was recorded by one nurse, and echocardiography was performed.

Measurement of QT was made in all possible leads, by one observer. Recordings were made by a digital ECG machine (Alicia Diagnostics, Sanford, FL, USA). The digitally recorded ECG tracings were evaluated using a digital clipper in Corel Photo Paint ver 13 software (Ottawa, Canada). Magnification of the ECG made it possible to determine the measurement points more precisely. The QT interval was measured from the beginning of the QRS complex to the termination of the T wave (defined as the return to the isoelectric line) in 12-lead ECG. Bazett’s formula was used to calculate the QTc

$Qt_{c}=\frac{\mathrm{QT}}{\sqrt[{2}]{R-\mathrm{R interval}}}$

The QT and QTc wave dispersion were then calculated according to the definition of dispersion—the difference between maximum and minimum duration values in 12-lead ECG. TPE interval was measured in each precordial lead. It obtained from the difference between QT interval and QT peak interval, measured from the beginning of the QRS to the peak of the T-wave. TPE dispersion was also calculated as the difference between the maximum and minimum TPE interval in precordial leads during a single beat.

Echocardiography was performed by a GE Vivid 3 echocardiographic machine (GE Vingmed, Horten, Norway) using a 3-MHz probe with pulsed Doppler tissue imaging software. All M-mode, two-dimensional, Doppler, and pulsed tissue Doppler echocardiographic studies were conducted with the patients in left lateral decubitus position by a one qualified cardiologist. The ejection fraction, shortening fraction, and septal and posterior wall thickness during systole and diastole were measured in the left parasternal long axis view. The pulsed Doppler sample volume was placed at the mitral valve and tricuspid tips, and three cardiac cycles were recorded from the apical window. Early (E) and late (A) peak velocities and their ratio were measured to assess the diastolic function. Pulsed tissue Doppler tissue images were obtained with the sample volume placed at the lateral corner of the mitral annulus, then at the medial (or septal) and tricuspid corner in the apical four-chamber view, and finally at the anterior wall and posterior wall in the parasternal short-axis view. In each region, systolic (S) wave, early diastolic (Ea), and late diastolic (Aa) wave velocities were recorded. The average of three consequtive waves was recorded, as well. 

Statistical analysis

SPSS^®^ for Windows^®^ ver 16.0 (SPSS Inc, Chicago, Il, USA) was used for data analyses. Continuous variables are expressed as mean±SD. The mean of normally distributed variables were compared among the study groups by one-way ANOVA. Pearson’s correlation coefficient was used to assess the level of correlation between serum electrolyte levels, echocardiographic indices recorded, and ECG parameters. A p value <0.05 was considered statistically significant.

## RESULTS

The mean±SD age of patients before transplantation, patients after transplantation, and normal age-matched children was 13.8±4.3, 13.2±4.8, 12.8±4.5 years, respectively. All the participants had a normal serum calcium, potassium, and sodium level. The mean±SD duration passed after the transplantation was 4.2±2.9 years. The post-transplantation patients were on tacrolimus or cyclosporine in addition to corticosteroids, mycophenolate mofetil, and azathioprine. The demographic data of study participants are presented in Table 1.

**Table 1:** Demographic data, serum electrolytes, and arterial blood gas parameters in the studied participants. Data are presented as mean±SD or ratio. The three groups were not significantly different in terms of the measured parameters

**Parameter**	Pre-transplantation	Post-transplantation	Control
Age (year)	13.8±4.3	13.2±4.8	12.8±4.5
Male/Female ratio	8/13	25/20	15/13
Weight (kg)	37.5±1.3	38.1±3.7	40.2±3.3
Blood urea nitrogen (mg/dL)	54.2±9.3	19.3±5.1	–
Serum creatinine (mg/dL)	5.2±0.8	1.09±0.32	–
Potassium (mEq/L)	4.2±0.6	4.4±0.4	–
Sodium (mEq/L)	140.8±3.2	140.6±2.3	–
Calcium (mg/dL)	9.1±0.2	9.2±0.4	–
Bicarbonate (mEq/L)	16.1±2.3	22.8± 2.1	–
Blood pH	7.32±0.13	7.4±0.2	–

A statistically significant difference was observed in the mean QT dispersion, QTc dispersion, and TPE dispersion among the three groups (Table 2).

**Table 2:** Comparison of ventricular repolarization indices in the studied participants. Values are mean±SD of indices in seconds. All the measured indices were significantly (p=0.0001) different among the study groups

Index	Pre-transplantation	Post-transplantation	Control
QT dispersion	0.083 ± 0.033	0.056± 0.030	0.034 ± 0.017
TPE dispersion	0.061±0.022	0.045 ±0.021	0.035±0.020
QTc dispersion	0.104±0.038	0.066±0.033	0.039±0.020

Left ventricular end-diastolic diameter was significantly correlated with QT dispersion (r = 0.35, p=0.01), QTC dispersion (r = 0.44, p=0.002), and TPE dispersion (r = 0.30, p=0.036). There was also a significant correlation between the systolic velocity of the lateral mitral annulus, which was measured by tissue Doppler, and TPE dispersion (r = 0.44, p=0.001). No significant correlation was observed between the ejection fraction; diameter of inter-ventricular septum; diameter of the left ventricular posterior wall; the mitral valve E wave and A wave velocities; E/A ratio; the tricuspid E wave and A wave velocities; S wave velocity of the lateral tricuspid valve annulus; Ea wave and Aa wave velocities of the lateral mitral valve annulus; Ea wave and Aa wave velocities of the lateral tricuspid valve annulus; E/Ea ratio of the mitral valve; and QT dispersion, QTc dispersion, and TPE dispersion.

## DISCUSSION

Cardiovascular events contribute to a significant proportion of all deaths in children with chronic renal disease. The European Dialysis and Transplant Association reported that 41% of all deaths in the children with ESRD were attributed to cardiovascular causes. In addition, the majority of the deaths were sudden and unexpected [13]. This mortality is often due to the higher incidence of the events such as arrhythmias, which can cause sudden death [7]. Therefore, finding a way to predict these lethal events is a topic of interest for research, especially with focusing on non-invasive methods such as ECG and echocardiography. 

A non-invasive way for assessing ventricular repolarization is the measurement of the QT interval. QT and QTc dispersion are useful indices for assessing depolarization changes and prediction of arrhythmias in ECG. When QT dispersion increases with heterogeneity in ventricular recovery time, it can be assumed that an increased QT dispersion would be an indicator of the disparity of the ventricular recovery time [14]. Some studies have shown that the risk of drug-induced arrhythmias (*eg*, torsade-de-pointes) is closely related to the enhancement of the normally existing transmural dispersion of repolarization rather than prolongation of the ventricular repolarization [15-17]. These studies showed a relationship between QT changes as well as repolarization time and arrhythmias.

Familoni, *et al*, indicated that the advantages of QT dispersion, as a non-invasive and useful method for evaluation of myocardial repolarization, cannot be over-emphasized [18]. The current study revealed that QT dispersion and QTc dispersion increased in end-stage renal disease (ESRD). Interestingly, these arrhythmogenic factors were improved after kidney transplantation. Similar findings have also been obtained by previous studies [19-22]. Morrison, *et al*, suggested that the increase in QT, QTc, and QT dispersion in patients with chronic renal failure might be non-invasive methods for detection of ventricular arrhythmias [24]. Brunner, *et al*, showed that QT dispersion was a powerful way for predicting ventricular arrhythmias and sudden death in patients with ESRD under chronic hemodialysis [25].

The effects of renal transplantation on these two ECG indices were evaluated by Koc, *et al*, who showed that the QT dispersion in renal transplant recipients was similar to that of the healthy control subjects [6]. On the contrary, we found that the QT dispersion improved after transplantation, but it did not return to its normal value. This difference may be due to various factors including age, time of transplantation, and pre-transplantation condition of these high risk patients.

TPE interval is also another predictor of ventricular arrhythmia though it has been mentioned less often in previous studies. Nevertheless, the role of increased TPE dispersion in ventricular arrhythmias has been confirmed by multiple studies [26, 27]. Tun, *et al*, described similar effect of ESRD on TPE dispersion as a trans-myocardial dispersion indicator in pre-dialysis patients with ESRD [28].

In the present study, only a significant correlation was observed between the left ventricular end-diastolic dimension and QT dispersion, QTc dispersion, and TPE dispersion. Tissue Doppler-derived systolic velocity of the lateral mitral valve annulus was also positively correlated with TPE dispersion. So far, a limited number of studies have addressed the correlation between echocardiographic parameters and ECG findings in transplanted children. Familoni, *et al*, showed a relationship between the left ventricular hypertrophy and the longest QTc dispersion [29]. However, the findings of the current study revealed no significant correlation between the ventricular wall dimension and changes in repolarization indices.

In conclusion we found that ESRD increases QT dispersion, QTc dispersion, and TPE dispersion in children. Renal transplantation improves these changes; however it does not return the indices to their normal values. The left ventricular internal dimension during the diastole also has a significant correlation with repolarization indices.
